# Comparing venetoclax in combination with hypomethylating agents to hypomethylating agent-based therapies for treatment naive *TP53*-mutated acute myeloid leukemia: results from the Consortium on Myeloid Malignancies and Neoplastic Diseases (COMMAND)

**DOI:** 10.1038/s41408-024-01000-2

**Published:** 2024-02-20

**Authors:** Talha Badar, Ahmad Nanaa, Ehab Atallah, Rory M. Shallis, Sacchi de Camargo Correia Guilherme, Aaron D. Goldberg, Antoine N. Saliba, Anand Patel, Jan P. Bewersdorf, Adam S. DuVall, Danielle Bradshaw, Yasmin Abaza, Guru Subramanian Guru Murthy, Neil Palmisiano, Amer M. Zeidan, Vamsi Kota, Mark R. Litzow

**Affiliations:** 1 Division of Hematology-Oncology, Mayo Clinic, Jacksonville, FL USA; 2 Division of Hematology, Mayo Clinic, Rochester, MN USA; 3 Department of Internal Medicine, John H. Stroger, Jr. Hospital of Cook County, Chicago, IL USA; 4 Division of Hematology and Medical Oncology, Medical College of Wisconsin, Milwaukee, WI USA; 5 Section of Hematology, Department of Internal Medicine, Yale School of Medicine, New Haven, CT USA; 6 Department of Internal Medicine, Mount Sinai Health System, New York, USA; 7 Division of Hematologic Malignancies, Department of Medicine Memorial Sloan Kettering Cancer Center, New York, NY USA; 8 Section of Hematology and Oncology, Department of Medicine, University of Chicago, Chicago, IL USA; 9 Division of Hematology and Oncology, Georgia Cancer Center, Augusta, GA USA; 10 Robert H. Lurie Comprehensive Cancer Center, Northwestern Hospital, Chicago, IL USA; 11 Division of Hematology and Oncology, Jefferson University Hospital, Philadelphia, PA USA

**Keywords:** Acute myeloid leukaemia, Epidemiology

## Abstract

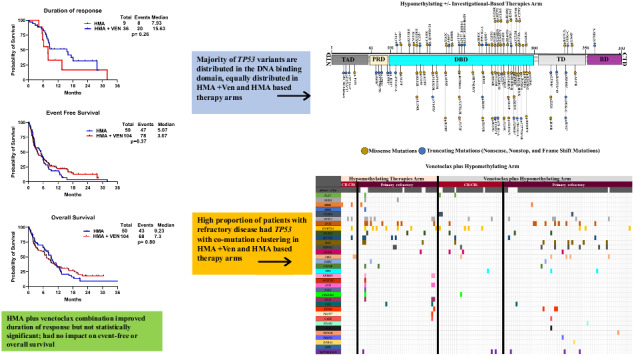

**Dear Editor,**

*TP53* is a tumor suppressor gene located on the short arm of chromosome 17(p13), known to be involved in multiple cellular processes and regulate cell proliferation by responding to various stress [1, 2]. *TP53* is a frequently mutated (m) gene found across diverse types of cancers, strongly linked to large structural and complex chromosomal abnormalities [3, 4]. It is reported in 5–10% of patients diagnosed with acute myeloid leukemia (AML), more prevalent among the elderly and those with therapy-related AML and/or complex cytogenetics (CG). AML with *TP53* mutations is associated with poor prognosis and inferior responses to traditional therapies [3, 4].

Venetoclax (VEN), is a potent and selective inhibitor of B-cell leukemia/lymphoma-2 (BCL-2), approved to treat newly diagnosed patients with AML that are unable to receive intensive chemotherapy [5, 6]. Responses to venetoclax and their durability are linked to specific molecular profiles, these associations are particularly notable with mutations in *DDX41, RUNX1, SRSF2, NPM1, IDH1* or *IDH2* [7–11]. Resistance to venetoclax-based combinations is frequently associated with the emergence of clones that impart resistance and is often linked to specific molecular signatures or pathways (*TP53*, *FLT3*, *DNMT3A* or *RAS*) [2, 8, 9].

Prior prospective and retrospective studies comparing HMA + VEN induction therapy to other approaches in *TP53*m AML did not demonstrate improved OS [4, 12]. This outcome might be attributed to the persistence of clones that did not undergo apoptosis during long-term VEN + HMA therapy, as previously documented [7]. In this work, we utilized a multi-institutional real-world database to explore the outcomes of VEN in combination with HMA compared to HMA-based treatment in a larger population of adult *TP53*m AML patients than previously reported.

The AML database of 381 patients with *TP53*m AML diagnosed between 2012 and 2022 was queried and 154 (40%) (HMA [*N* = 50; 32%] and HMA + VEN [*N* = 104; 68%]) patients were eligible for this analysis. The study conducted after obtaining approval from the Institutional Review Board (IRB), adhering to the ethical standards of the Declaration of Helsinki of 1975, as revised in 2000. Details on diagnosis, response assessment and statistical analysis provided in (Supplementary methods). Bi-allelic *TP53*m was defined by the presence of (1) 2 or more distinct *TP53*m with variant allele frequency (VAF) > 10% or a single *TP53*m associated with (i) complex cytogenetic (CG) abnormalities (ii) involving chromosome 17p (e.g., abnormality of 17p or monosomy 17) or (iii) single *TP53*m with a VAF of ≥50% [13].

The median age at diagnosis was 74 (38–87) for the HMA group compared to 71 (29–88) for the HMA + VEN group. In the HMA group, 34 (68%) and in the HMA + VEN group, 57 (55%) patients were aged 70 years or older (*p* = 0.16). Characteristics and hematological features of treatment naive *TP53*m AML patients undergoing treatment with HMA + VEN vs. HMA, summarized in Supplementary Table 1. Secondary (s) AML (evolving from prior myelodysplasia, and/or myeloproliferative neoplasm [MPN]) was observed in 26% of patients in the HMA group, compared to 31% in the HMA + VEN group (*p* = 0.6); 2 (4%) and 5 (5%) patients had prior history of MPN progress to AML (MPN blast phase) in HMA and HMA+Ven group, respectively (*p* ≥ 0.99). Complex cytogenetics (CG) found in 86% of patients in the HMA group and in 90% of the HMA + VEN group (*p* = 0.6). Regarding *TP53* mutation status, 68% of patients in the HMA group had bi-allelic/multi-hit *TP53*m (MH *TP53*) compared to 73% in the HMA + VEN group (*p* = 0.6). The proportion of patients with frequently (≥5%) occurring myeloid co-mutations (*RUNX1, ASXL1, TET2, DNMT3A, RAS, and PTPN11*) did not demonstrate a significant difference between the HMA and HMA + VEN groups. Additionally, the co-mutation pattern did not predict outcomes within our cohort, as indicated in (Supplementary Table 2). Supplementary Fig. 1 provides an overview of the *TP53* protein structure, highlighting its domains and presenting the distribution of detected variants in our study based on different treatment options (HMA vs HMA + VEN).

Complete remission rates were higher in the HMA + VEN group compared to the HMA-based treatment group (35% vs. 18%, *p* = 0.05). Similarly, the proportion of responding patients receiving allogeneic stem cell transplants (allo-HCT) after induction was higher in the HMA + VEN group compared to the HMA group (13% vs. 4%; *p* = 0.14). Twenty-nine (19%) patients died in first 30 days post induction; 3% in HMA vs 25% in HMA + VEN group (*p* = 0.03). Among these 29 patients, 18 (HMA + VEN [*n* = 15] and HMA [*n* = 3]), 5 (all in HMA + VEN group) and 4 (all in HMA + VEN group) patients died from infection, bleeding, and tumor lysis syndrome, respectively. Details on causes of death summarized in Supplementary Material. In Fig. 1 we have illustrated co-mutation patterns, and cytogenetic status among sub-group of patients who achieved CR/CRi and those who had primary refractory disease.

**Fig. 1:**
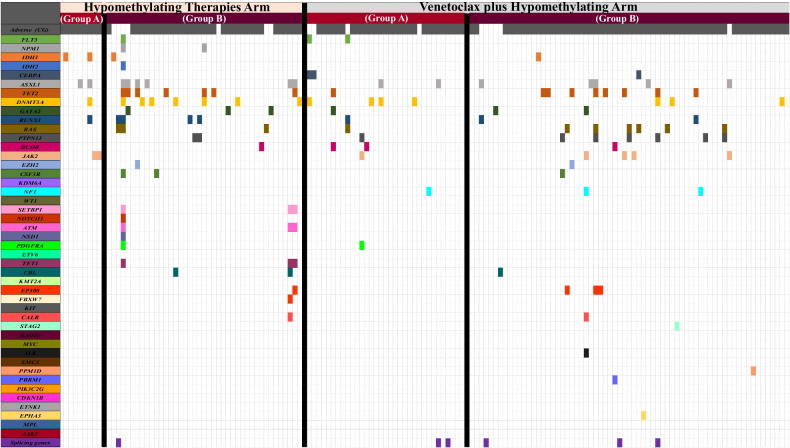
Co-Mutation Patterns & adverse cytogenetic findings observed in the *TP53* Cohort. This figure illustrates the patterns of co-mutations identified in the *TP53* cohort, categorized by treatment arm. Additionally, the data was further stratified based on two groups: the patients who achieved CR/CRi (**A**) and the primary refractory group (**B**).

The median duration of response (DOR) was higher in the HMA + VEN group (15.6 months) compared to the HMA group (7.93 months), but the difference was not statistically significant (*p* = 0.26) (Fig. 2A). The median follow up of the entire cohort was 6.5 months (range, 0.3–55.7). The median event free survival (EFS) and the median OS did not significantly differ between the HMA and HMA + VEN groups; (5.07 vs. 3.67 months, *p* = 0.37) and (9.23 vs. 7.3 months, *p* = 0.80) respectively (Fig. 2B, C).

**Fig. 2:**
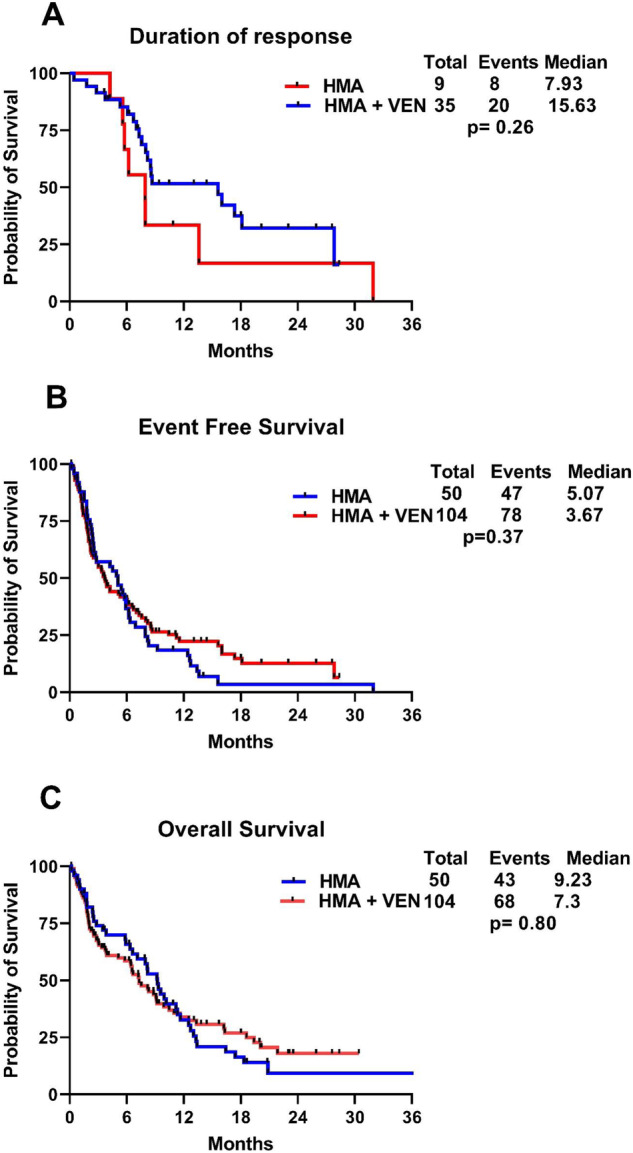
Kaplan–Meier survival curves by treatment regimens. This figure illustrates Kaplan–Meier curves of treatment-naïve mutated *TP53* AML patients, stratified by the treatment received—HMA + VEN vs. HMA: **A** Median Duration of Response. **B** Event-Free Survival. **C** Overall Survival.

We performed a multivariate analysis (MVA) for EFS and OS using variables that showed significance in univariate analysis (*p* < 0.05) (Supplementary Table 2). In MVA, the achievement of CR/CRi retained favorable significance for OS (HR: 0.36, 95% CI: 0.22–0.59 *p* < 0.001) but not for EFS (HR: 0.80, 95% CI: 0.18–3.52, *p* = 0.77). Similarly, allo-HCT in responding (CR/CRi) patients maintained a favorable significance for EFS (HR 0.06, 95% CI: 0.09–0.50, *p* = 0.009) and OS (HR 0.10, 95% CI: 0.14–0.76, *p* = 0.02) in MVA. Complex cytogenetics retained significance for shorter OS in MVA (HR: 2.47, 95% CI: 1.20–4.81, *p* = 0.01).

In this multi-institutional real-world study conducted across academic institutions in the US over a span of 10 years, while we have observed improvements in CR rates (35% vs. 18%), an extended median duration of response (15.6 vs. 7.93 months), and a higher proportion of patients progressing toward allo-HCT (13% vs. 4%) with HMA + VEN compared to HMA based treatment, this did not translate in an improvement in OS. Furthermore, no significant differences observed in baseline characteristics, co-mutation patterns, or the presence of complex cytogenetics and MH *TP53*m among the two groups.

In a recent pooled analysis of two large trials (NCT02993523 and NCT02203773), 54 patients diagnosed with poor-risk cytogenetics and *TP53*m AML treated with HMA + VEN were compared to 18 patients treated with HMAs [5, 14]. The baseline characteristics and proportion of patients with sAML were similar to those in our analysis. Among patients with *TP53*m AML from the pooled analysis, the median duration of response (DOR) was 6.5 months for HMA + VEN and 6.7 months for HMAs alone. Similarly, patients had a poor median OS regardless of the treatment approach; 5.2 months for HMA + VEN and 4.9 months for HMAs alone. These findings align with our observations, where we did not observe a survival advantage with the addition of venetoclax to HMA-based therapy. The majority of trials and retrospective reports in the literature on HMA + VEN outcomes in treatment naïve *TP53*m AML, have reported CR rates of approximately 20–40% and median OS range of (6.0–11.0 months) [5, 9]. In recently conducted real-world study on 301 AML patients, who were treated with HMA + VEN, *TP53*m was deemed unfavorable with inferior CR rates and overall survival [8]. The data suggest desperate need for novel and effective treatment combinations for patients with *TP53*m AML.

Although HMA + VEN combination is approved to managed elderly AML patients who are ineligible for intensive chemotherapy, tolerance of this combination in elderly frail patients can be challenging with prolong myelosuppression, infections and increase mortality, especially with continuous dosing of venetoclax. Based on our observation, *TP53*m AML patients who are frail with sub-optimal performance status can be manage with HMA based therapy alone without compromising their survival or on a reduced duration of venetoclax with HMA based on recent data that suggest no significant benefit in survival with continuous 28 days of venetoclax compared to 21 days or 14 days of venetoclax per cycle [10].

While allo-HCT is considered a potentially curative option for high-risk AML, there are conflicting reports regarding its utility in improving the survival of patients with *TP53*m AML. The lack of benefit was frequently attributed to the difficulty in achieving a complete response and the persistence of the *TP53-*mutated clone before undergoing allo-HCT [7]. In our study, among patients with CR/CRi who underwent allo-HCT, regardless of HMA or HMA + VEN induction showed improved OS, as have been reported previously [3].

In conclusion, there is a dire need for the development of more effective treatment strategies that are less susceptible to resistance to improve outcomes of patients with *TP53*m AML. Progress in immunotherapeutics and approaches targeting mutant *TP53* protein may offer promise in improving outcomes for patients diagnosed with *TP53*m AML [4]. We acknowledge the limitations associated with the retrospective design of our research and the inherent selection bias. Despite these challenges, our data presents the first real-world insights from a substantial multi-center patient cohort with *TP53*m AML, comprehensively analyzed via NGS and closely monitored longitudinally.

### Supplementary information

Supplementary Material
