# Supplementary material for: Comparing venetoclax in combination with hypomethylating agents to hypomethylating agent-based therapies for treatment naive TP53-mutated acute myeloid leukemia: results from the Consortium on Myeloid Malignancies and Neoplastic Diseases (COMMAND)
Source: Blood Cancer J. 2024 Feb 20;14(1):32. doi: 10.1038/s41408-024-01000-2 (PMC10879201; doi:10.1038/s41408-024-01000-2)
Supplement: Supplementary file 1 — Supplementary Material [file 41408_2024_1000_MOESM1_ESM.docx]

**Comparing Venetoclax in Combination with Hypomethylating Agents to Hypomethylating Agent-Based Therapies for** **Treatment Naive *TP53*-Mutated Acute Myeloid Leukemia: Results from the Consortium on Myeloid Malignancies and Neoplastic Diseases (COMMAND)**

Talha Badar, MD^1^ Ahmad Nanaa, MD^1,2,3^, Ehab Atallah, MD^4^, Rory M. Shallis, MD^5^, Sacchi de Camargo Correia Guilherme, MD^6^, Aaron D. Goldberg, MD, PhD^7^, Antoine N. Saliba, MD^2^, Anand Patel, MD^8^, Jan P. Bewersdorf, MD^7^, Adam S DuVall, MD^9^, Danielle Bradshaw, MD^9^, Yasmin Abaza, MD^10^, Guru Subramanian Guru Murthy, MD^4^, Neil Palmisiano, MD^11^, Amer M. Zeidan, MD^5^, Vamsi Kota, MD^9^, Mark R. Litzow, MD^2^

^1^Division of Hematology-Oncology, Mayo Clinic, Jacksonville, FL, USA.

^2^Division of Hematology, Mayo Clinic, Rochester, MN, USA

^3^Department of Internal Medicine, John H. Stroger, Jr. Hospital of Cook County, IL, USA.

^4^Division of Hematology and Medical Oncology, Medical College of Wisconsin, Milwaukee, WI, USA

^5^Section of Hematology, Department of Internal Medicine, Yale School of Medicine, New Haven, CT, USA.

^6^Department of Internal Medicine, Mount Sinai Health System, New York, USA

^7^Division of Hematologic Malignancies, Department of Medicine Memorial Sloan Kettering Cancer Center, NY, USA.

^8^Section of Hematology and Oncology, Department of Medicine, University of Chicago, Chicago, IL, USA.

^9^ Division of Hematology and Oncology, Georgia Cancer Center, GA, USA.

^10^Robert H. Lurie Comprehensive Cancer Center, Northwestern Hospital, Chicago, Illinois.

^11^Division of Hematology and Oncology, Jefferson University Hospital, Philadelphia, PA, USA

**Supplemental methods**

This study is a retrospective, multi-Institutional analysis conducted through COMMAND (The Consortium on Myeloid Malignancies and Neoplastic Diseases), a collaboration of acute leukemia experts from ten academic institutions in the United States (US). It investigated the outcomes of treatment naïve AML patients with pathogenic/likely pathogenic (P/LP) *TP53* mutations (m) who received combination therapy with HMA+VEN compared to HMA-based therapies in adults aged 18 and above. The study spanned from 2012 to 2022 and was conducted after obtaining approval from the Institutional Review Board (IRB). The procedures were conducted in compliance with the Declaration of Helsinki of 1975, as revised in 2000.

Treatment response was evaluated using the 2017 European Leukemia Net (ELN) consensus guidelines^1^. At the time of diagnosis, Next-Generation Sequencing (NGS) was performed using DNA extracted from bone marrow aspirate specimens. Post-sequencing analysis was then carried out to identify mutations associated with myeloid neoplasms. The development of NGS testing and the determination of its performance characteristics were carried out by the participating institutions, adhering to the requirements of the Clinical Laboratory Improvement Amendments (CLIA)^2, 3^.

**Statistical analyses**

All analyses were conducted using IBM^®^ SPSS^®^ software. Categorical parameters were compared using the Chi-square test (χ2), while the Wilcoxon test was employed for continuous variables. Median overall survival (OS) was calculated from the time of diagnosis to the occurrence of death or the last follow-up. Event-free survival (EFS), defined as the time from diagnosis to relapse or death, was estimated using the Kaplan-Meier method. Univariate and multivariate predictors of overall mortality and progression were determined using Cox proportional hazards regression models. Multivariable models included all significant univariate predictors. Significance was determined using two-sided tests, with a p-value of <0.05 considered statistically significant.

**Supplementary Data**

*Causes of death*

One hundred eleven (72%) died till last follow up; 48 (43%) patients from refractory leukemia, 30 (27%) patients from infections, 5 (4.5%) patients from bleeding, 4 (4%) patients from tumor lysis syndrome (TLS), 3 (3%) patients from respiratory or cardiac failure and for 21 (19%) patients reason of death was not documented. Twenty-nine (19%) patients died in first 30 days post induction; 3% in HMA vs 25% in HMA+Ven group (p= 0.03). Among these 29 patients, 18 (HMA+Ven [n=15] and HMA [n=3]), 5 (all in HMA+Ven group) and 4 (all in HMA+Ven group) patients died from infection, bleeding, and tumor lysis syndrome, respectively.

**Supplementary Table 1:** Characteristics and hematological features of treatment naive *TP53*-mutated acute myeloid leukemia patients undergoing treatment with hypomethylating agents with or without investigational therapies, and patients treated with a combination of venetoclax and hypomethylating agents.

| **Variables** | **HMA +/- invetigational agent*** | **HMA + VEN** | **p value** |
| --- | --- | --- | --- |
| **No. of patients, (%)** | 50 (32) | 104 (68) |  |
| **Age in years (median)** | 74 [38-87] | 71 [29-88] | 0.23 |
| **Age > 70 years, n (%)** | 34 (68) | 57 (55) | 0.16 |
| **Gender (male), n (%)** | 24 (48) | 66 (64) | 0.08 |
| **Secondary AML, n (%)** | 13 (26) | 32 (31) | 0.57 |
| **MPN blast phase** | 2 (4) | 5 (5) | >0.99 |
| **Therapy related, n (%)** | 18 (36) | 31 (30) | 0.46 |
| **WBC (10^9^/L)** | 2.36 [0.56-96.5] | 2.63 [0.40-460] | 0.86 |
| **BM blast (%)** | 30 [12-90] | 32 [7-99] | 0.28 |
| **PB blast (%)** | 8 [0-90] | 14 [0-92] | 0.63 |
| **Complex CG, n (%)** | 43 (86) | 94 (90) | 0.58 |
| **Multi-hit *TP53*, n (%)** | 34 (68) | 76 (73) | 0.56 |
| **Co-mutations (> 5% overall), n (%)** |  |  |  |
| ***RUNX1*** | 5 (10) | 3 (3) | 0.10 |
| ***ASXL1*** | 8 (16) | 7 (7) | 0.07 |
| ***TET2*** | 7 (14) | 9 (9) | 0.26 |
| ***DNMT3A*** | 8 (16) | 6 (6) | 0.06 |
| ***RAS*** | 3 (6) | 7 (7) | >0.99 |
| ***PTPN11*** | 2 (4) | 7 (7) | 0.72 |
| **Complete remission (CR or CRi), n (%)** | 9 (18) | 36 (35) | 0.05 |
| **30 days post-induction mortality** | 3 (6) | 26 (25) | 0.03 |
| **Allo-HCT, n (%)** | 2 (4) | 13 (13) | 0.14 |
| **HMA; hypomethylating agent, VEN; venetoclax, CG; cytogenetics, BM; bone marrow, allo-HCT; allogeneic stem cell transplantation**  ***** **8 (16%) patients received investigational agents combinations including entospletinib (n=3), glasdegib (n=2), inecalcitol (n=2) and durvalumab (n=1).** | | | |

**Supplementary Table 2:** Predictors of event-free and overall survival in treatment naive AML patients with *TP53* mutations who received combination therapy with HMA+VEN or HMA based therapy.

| **Variables** | **Event free survival**  **Univariate**  ***P* value**  **Hazard ration (95% CI)** | **Event free survival**  **Multivariate**  ***P* value**  **Hazard ration (95% CI)** | **Overall Survival**  **Univariate**  ***P* value**  **Hazard ration (95% CI)** | **Overall Survival**  **Multivariate**  ***P* value**  **Hazard ration (95% CI)** |
| --- | --- | --- | --- | --- |
| **Age > 70 years** | 0.12  1.34 (0.94-1.91) | **-** | 0.62  1.25 (0.85-1.82) | **-** |
| **Gender (male)** | 0.89  1.02 (0.72-1.45) | **-** | 0.58  1.11 (0.76-1.61) | **-** |
| **Secondary AML** | 0.52  1.13 (0.76-1.66) | **-** | 0.95  0.98 (0.65-1.48) | **-** |
| **Therapy related** | 0.18  0.77 (0.53-1.11) | - | 0.09  0.71 (0.48-1.04) | - |
| **Complex CG** | 0.48  1.20 (0.72-1.99) | - | **0.03**  **0.52 (0.32-0.86)** | 0.01  2.47 (1.20-4.81) |
| **Multi-hit *TP53*** | 0.57  0.89 (0.60-1.32) | - | 0.88  0.97 (0.64-1.45) | - |
| **Co-mutations (> 5% overall)** |  |  |  |  |
| ***RUNX1*** | 0.88  0.94 (0.47-1.90) | - | 0.24  0.61 (0.31-1.20) | - |
| ***ASXL1*** | 0.92  1.03 (0.56-1.88) | - | 0.84  1.06 (0.55-2.01) | - |
| ***TET2*** | **0.02**  **1.83 (0.91-3.65)** | 0.33  2.75 (0.34-21.8) | 0.057  1.74 (0.84-3.59) | - |
| ***DNMT3A*** | 0.33  0.74 (0.42-1.28) | - | 0.65  0.85 (0.45-1.62) | - |
| ***RAS*** | 0.09  1.69 (0.75-3.83) | - | 0.44  1.32 (0.58-2.97) | - |
| ***PTPN11*** | 0.13  1.66 (0.71-3.90) | - | 0.06  1.93 (0.73-5.06) | - |
| **Complete remission (CR or CRi)** | **<0.001**  **0.27 (0.19-0.39)** | 0.77  0.80 (0.18-3.52) | **<0.001**  **0.29 (0.20-0.43)** | <0.001  0.36 (0.22-0.59) |
| **Allo-HCT among patients who achieved CR or CRi** | **0.0002**  **0.15 (0.07-0.32)** | **0.009**  **0.06 (0.09-0.50)** | **0.004**  **0.16 (0.06-0.40)** | 0.027  0.10 (0.14-0.76) |

**
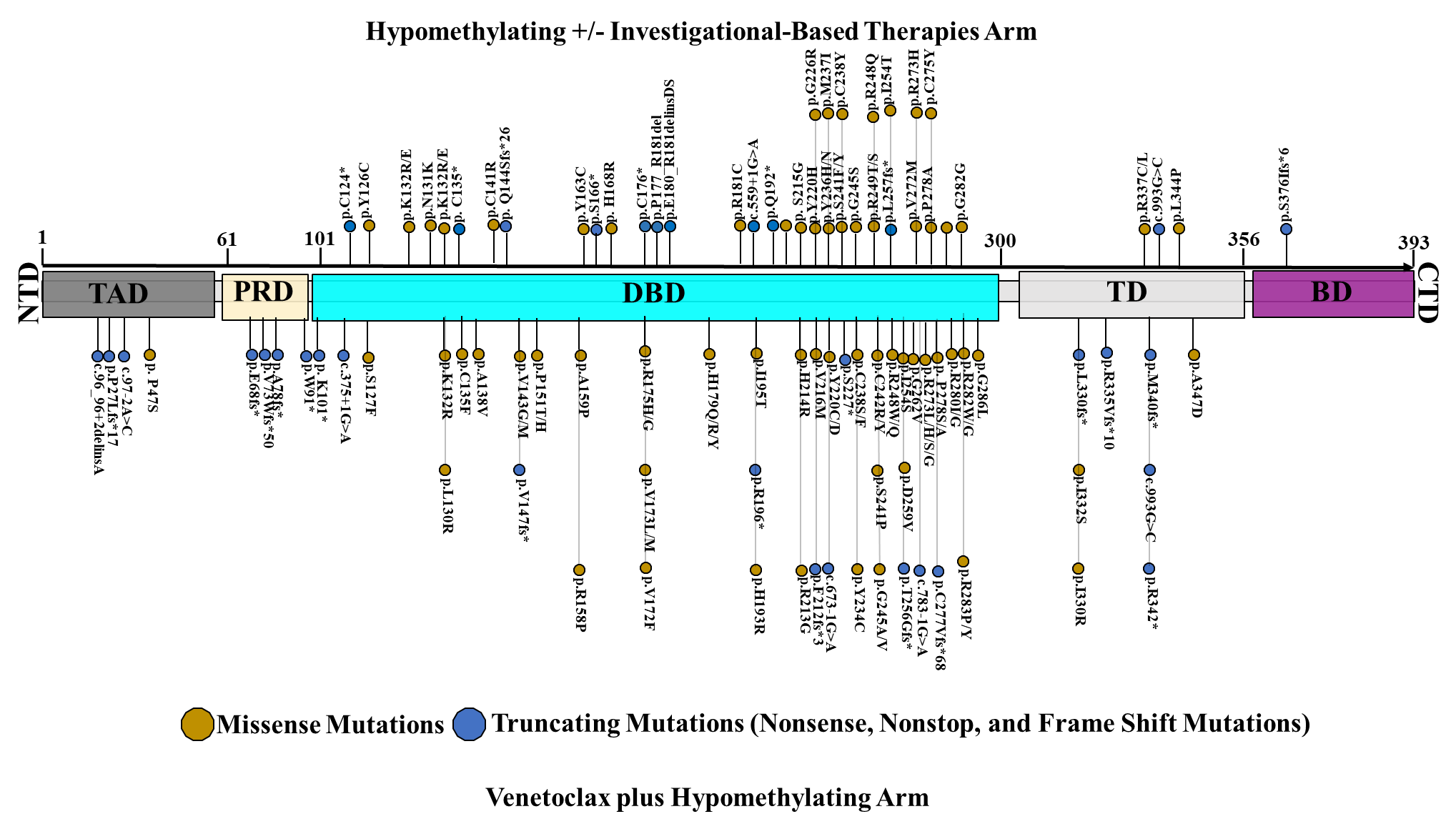
Supplementary Figure 1:** This figure represents an overview of *TP53* domains, structures, and the distribution of *TP53* variants positioned on the *TP53* protein. Variants identified in patients treated with hypomethylating therapies are depicted at the top, while those from patients treated with venetoclax plus hypomethylating agents are shown at the bottom. Variants are represented as gold circles for missense mutations, and as blue circles for all other variant types, including truncated mutations corresponding to splice site variants, nonsense, nonstop, and frameshift deletions or insertions. **Key:** NTD: N-terminal domain, TAD: Transactivation domain, PRD: Proline-rich domain, DBD: DNA-binding domain, TD: Tetramerization domain, BD: Basic domain, CTD: C-terminal domain.

**References**

1. Döhner H, Estey E, Grimwade D, Amadori S, Appelbaum FR, Büchner T, et al. Diagnosis and management of AML in adults: 2017 ELN recommendations from an international expert panel. Blood. 2017;129(4):424-47.

2. Badar T, Atallah E, Shallis R, Saliba AN, Patel A, Bewersdorf JP, et al. Survival of TP53-mutated acute myeloid leukemia patients receiving allogeneic stem cell transplantation after first induction or salvage therapy: results from the Consortium on Myeloid Malignancies and Neoplastic Diseases (COMMAND). Leukemia. 2023;37(4):799-806.

3. Badar T, Atallah E, Shallis RM, Goldberg AD, Patel A, Abaza Y, et al. Outcomes of TP53-mutated AML with evolving frontline therapies: Impact of allogeneic stem cell transplantation on survival. American Journal of Hematology. 2022;97(7):E232-E5.
